# Which factors play a role in Dutch health promotion professionals’ decision to recruit actively primary schools to use a web-based smoking prevention programme?

**DOI:** 10.1186/1756-0500-6-504

**Published:** 2013-12-03

**Authors:** Henricus-Paul Cremers, Anke Oenema, Liesbeth Mercken, Math Candel, Hein de Vries

**Affiliations:** 1 Department of Health Promotion, School for Public Health and Primary Care (CAPHRI), Maastricht University, P.O. Box 616, 6200 MD Maastricht, The Netherlands; 2 Department of Methodology and Statistics, School for Public Health and Primary Care (CAPHRI), Maastricht University, P.O. Box 616, 6200 MD Maastricht, The Netherlands

**Keywords:** Recruitment, Decision-making, Smoking prevention, Primary school

## Abstract

**Background:**

Municipal Health Promotion Organisations (MHPOs) play an important role in promoting and disseminating prevention programmes, such as smoking prevention programmes, in schools. This study identifies factors that may facilitate or hinder MHPOs’ willingness to recruit actively primary schools to use a smoking prevention programme.

**Methods:**

In 2011, 31 Dutch MHPOs were invited to recruit schools to use a smoking prevention programme. All MHPO employees involved in smoking prevention activities (*n* = 68) were asked to complete a questionnaire assessing psychological factors and characteristics of their organisation that might affect their decision to be involved in active recruitment of schools. *T*-tests and multivariate analysis of variance assessed potential differences in psychological and organisational factors between active and non-active recruiters.

**Results:**

A total of 45 professionals returned the questionnaire (66.2%). Active recruiters (*n* = 12) had more positive attitudes (*p* = 0.02), higher self-efficacy expectations (*p* < 0.01) and formulated more plans (*p* < 0.01) to recruit primary schools, compared with non-active recruiters. Organisational factors did not discriminate between active and non-active recruiters.

**Conclusions:**

Primarily psychological factors seem to be associated with MHPOs’ decision to recruit schools actively. This indicates that creating more positive attitude, self-efficacy beliefs and formation of plans may help in getting more MHPOs involved in active recruitment procedures.

## Background

Smoking (experimentation) among youngsters remains a public health problem [1,2]. Although smoking levels among primary school pupils are low, this percentage increases rapidly (21% at age 15) when children make the transition to secondary school [3]. Smoking prevention programmes implemented in primary schools have the potential to be effective in preventing the uptake of smoking when children make the transition from primary to secondary school [4]. Since addiction can already occur after smoking only a few cigarettes [5,6], there is a need for smoking prevention programmes targeting primary school children [7]. This requires that effective smoking prevention programmes are available and that primary schools implement programmes in their curriculum to prevent smoking uptake of children, when they transit from primary to secondary school.

In order to foster the utilisation of smoking prevention programmes by primary schools, an active approach in reaching schools is considered to be the most effective [8]. Municipal Health Promotion Organisations (MHPOs) in the Netherlands function as an intermediary organisation and are legally responsible for promoting and disseminating smoking prevention programmes to primary schools [9-11]. However, MHPOs often face difficulties (e.g. time constraints or prevention programmes are not in line with school policies) in disseminating prevention programmes to schools [12] and, as a result, implementation of programmes through such intermediary organisations is not always optimal [13]. To be able to optimise the process of programme dissemination to schools, it is important to identify which factors may hinder or facilitate the decision of intermediary organisations, such as MHPOs, to participate in the active promotion of (smoking) prevention programmes to schools.

Dissemination, a planned and systematic process to make programmes widely available, is an important and well-known aspect of diffusion strategies, which further include adoption, implementation and maintenance [14]. However, in order to adopt and implement programmes by primary schools, intermediary organisations should consider whether they are willing to fulfil a role in the dissemination process. Previous studies have identified a variety of factors that may influence the dissemination of a product or programme in an organisation [15-18], such as characteristics of the organisational context or the decision-making style (e.g. centrality, formalisation, information and confrontation) [18-22]. Besides these organisational factors, dissemination of a new product or programme may be influenced by psychological factors, such as attitude, social influence, self-efficacy expectations and preparing for action by the decision-making individual (i.e. a person who is responsible for decisions within an organisation) [20,23].

In line with previous studies that have investigated dissemination and implementation processes, this study aims to assess which psychological and organisational factors are associated with MHPO employees’ willingness to participate in the active recruitment of Dutch primary schools to use a smoking prevention programme.

## Methods

### Study design and procedure

In October-November of 2010, health promotion professionals (HPPs) responsible for smoking prevention in the primary schools of all MHPOs (*n* = 31) in the Netherlands were invited by telephone and e-mail to participate in the active recruitment of primary schools to use the smoking prevention programme ‘Fun without Smokes’ [24]. The ‘Fun without Smokes’ programme is a web-based computer tailored smoking prevention programme that will be evaluated in a cluster randomised controlled trial (c-RCT). Participation in the active recruitment of schools included approaching and informing primary schools about the smoking prevention programme that could be implemented in the last two grades of primary school (children aged 10 – 12 years) and motivating them to use the programme and participate in the evaluation study.

At the beginning of 2011, all contacted HPPs (*n* = 68) who were involved in the decision, whether or not to participate in the active recruitment of primary schools (decision makers), were asked to take part in a cross-sectional study. HPPs had to fill out a print-delivered questionnaire, containing questions concerning attitudes, social influences, self-efficacy expectations, preparatory plans and the decision-making style of the MHPO. HPPs were able to indicate whether they preferred receiving the questionnaire through e-mail or postal mail. All participants received an information brochure that explained the goals, procedures and background of the ‘Fun without Smokes’ programme, the evaluation study and the current dissemination study and questionnaire. HPPs were instructed to complete the questionnaire before they started with the recruitment of primary schools. After completion, all participants were able to return the questionnaire through e-mail or postal mail without any costs.

The primary outcome measure in the present study was active or non-active recruitment of primary schools for the ‘Fun without Smokes’ programme. Active recruitment involved contacting schools through telephone or visiting schools to inform them about the goals of the ‘Fun without Smokes’ programme. Non-active recruitment was used to classify MHPOs who decided not to participate in the recruitment procedure.

### Measurement

*Demographics* measured were gender (1 = male; 2 = female), age (in years), smoking status (1 = yes, daily; 2 = yes, sometimes; 3 = no, but I used to smoke; 4 = no, I have never smoked), position in MHPO (1 = health promotion officer; 2 = health promotion employee; 3 = researcher/epidemiologist; 4 = quality coordinator; 5 = management function; 6 = other function), hours of employment (hours a week), experience in the field of smoking prevention (in years), experience as a HPP (in years) and total work experience (in years).

*Behaviour* was reported by every MHPO when they had the final decision whether or not to participate in the active recruitment of schools. This decision was reported to the research team by telephone or e-mail. Active recruitment was validated by a web-based database in which MHPOs registered which schools they had contacted and which schools had decided to participate. This procedure was verified by the researcher. Active recruitment of the MHPO was scored with a ‘1’ and non-active recruitment with a ‘0’.

### Psychological factors

*Attitude* towards offering smoking prevention programmes to schools in general and the ‘Fun without Smokes’ programme in particular were measured with 10 questions. Examples of questions were ‘I think it is important to offer smoking prevention programmes to primary schools’ or ‘Do you think the Fun without Smokes programme is an effective smoking prevention programme for primary school children?’ These questions could be scored on a five-point Likert scale, ranging from +2 = ‘totally agree’ to −2 = ‘totally disagree’ or +2 = ‘definitely yes’ to −2 = ‘definitely not’ (Cronbach’s alpha = 0.63).

*Social influence* was measured by 13 social support questions. HPPs had to indicate whether they expected to receive support from potentially important people in their professional environment such as teachers, head of department or colleagues. Other questions measured the social support concerning ‘Fun without Smokes’, such as: ‘Do you think primary schools in your region support the adoption of Fun without Smokes?’ These questions could be scored on a five-point Likert scale ranging from +2 = ‘definitely yes’ to −2 = ‘definitely not’ (Cronbach’s alpha = 0.89).

*Self-efficacy expectations* towards offering smoking prevention programmes to schools were measured by 12 questions on an answering scale ranging from +2 = ‘definitely yes’ to −2 = ‘definitely not’. ‘Are you able to offer a smoking prevention programme to primary schools in an active manner?’, ‘Are you able to convince primary schools of the importance of smoking prevention programmes?’ and ‘Are you able to recruit schools for the Fun without Smokes programme?’ are examples of questions used to measure self-efficacy expectations concerning smoking prevention programmes and ‘Fun without Smokes’ (Cronbach’s alpha = 0.93).

*Preparatory plans* included several steps for getting information about the ‘Fun without Smokes’ programme and planning for how and when to approach primary schools. A total of eight questions measured whether the HPPs would formulate plans; this was scored on a five-point Likert scale (+2 = ‘definitely yes’ to −2 = ‘definitely not’). An example of a possible preparatory plan was: ‘I am planning to visit the Fun without Smokes website’ (Cronbach’s alpha = 0.92).

### Organisational factors

*Centrality* towards decision-making is the extent to which top management involves lower levels in the decision-making process. This dimension was measured with one question: ‘Does your superior involve subordinates when a decision has to be taken whether or not to participate in the active recruitment of primary schools to use a prevention programme?’

*Formalisation* is the extent to which the decision-making process follows standard or more informal procedures. Formalisation was measured by the following question: ‘Do you follow formal procedures (standards or protocols) if a decision has to be taken as to whether or not to participate in the active recruitment of primary schools?’

*Information* measured whether the decision was based on the provided information and a consideration of pros and cons: ‘Do you weigh the pros and cons of the available information if a decision has to be taken whether or not to participate in the active recruitment of primary schools?’

*Confrontation* within decision-making is the extent to which decisions are the result of a political process in which a manager has to confront other parties that have opposing interests. Confrontation was measured by: ‘Does your superior confront colleagues with conflicting interests if a decision has to be taken whether or not to participate in the active recruitment of primary schools?’ All four questions to measure the decision-making style of an organisation could be scored on a five-point Likert scale, ranging from +2 = ‘totally agree’ to −2 = ‘totally disagree’ [18,19,22] and were analysed as individual items.

*Decision power* of each HPP was measured with one question: ‘How big was your share in the decision whether or not to take part in the active recruitment of primary schools with the smoking prevention programme Fun without Smokes?’ HPPs were able to indicate the percentage of their participation on a categorical scale: 1 = 0-24%; 2 = 25-49%; 3 = 50-74% or 4 = 75-100%.

*The amount of time available for smoking prevention* was measured as: ‘Do you have enough time to offer smoking prevention programmes to primary schools?’ A five-point Likert scale was used to score this question: +2 = ‘enough time’ to −2 = ‘not enough time’.

### Ethical approval

The present study is part of a larger smoking prevention intervention study called ‘Fun without Smokes’ [24]. The design, procedure and content of the ‘Fun without Smokes’ study is approved by the Medical Ethics Committee of the Atrium-Orbis-Zuyd Hospital (NL32093.096.11/MEC 11-T-25) and registered in the Dutch Trial Register (NTR3116). Participants in the present study were contacted and informed about the study via telephone by the researcher. After explaining the details of the study to the HPPs, verbal consent was requested whether or not they wished to be involved in the present study.

### Analyses

First, general descriptives were used to evaluate differences in demographic characteristics of active and non-active recruiters. Additionally, *t*-tests were performed to assess whether there were differences between active and non-active recruiters in psychological and organisational factors. Multivariate analysis of variance was used to calculate eta-squared (η^2^) [25], which assesses the strength of the relation between active versus non-active recruiters, on the one hand, and psychological and organisational factors, on the other. In logistic regression analysis no comparable measure is available to calculate the strength of effects between psychological and organisational factors. For the analyses the statistical software package SPSS 17.0 was used. To assess the difference between η^2^ of psychological and organisational factors, bootstrapping was used to calculate a bias-corrected accelerated confidence interval (BCa-CI) [26] of the difference in η^2^. When the confidence interval does not include 0, this indicates that either psychological or organisational factors might be important in the decision to participate in the active recruitment of primary schools. The confidence interval was obtained in R, programme version 1.3-4 [27].

## Results

### Basic characteristics

MHPOs varied in the number of decision makers that were involved in the active recruitment of schools, ranging from one to five HPPs per MHPO (average = 2.2 HPPs). Of the 31 MHPOs that were approached seven (22.6%) decided to recruit schools actively. They informed primary schools via telephone calls or school visits and stimulated them to participate in the ‘Fun without Smokes’ study. The residual 24 MHPOs decided not to take part in the active recruitment.

In the current study, a total of 45 HPPs (66.2%) returned the questionnaire. Of those 45 HPPs, 12 active recruiters (80%) returned the questionnaire, whereas 33 non-active recruiters (62.3%) responded. The majority of the HPPs who returned the questionnaire were female (80%) with a mean age of 41.6 years. Gross of the decision makers was a health promotion officer in both active (75%) and non-active recruiters (45.5%). Of the non-active recruiters two HPPs were smokers (6.3%), whereas of the active recruiters no one smoked. Additionally, more active recruiters indicated having maximum decision power (50%) compared with non-active recruiters (33.3%). As shown in Table 1, there were no significant differences between active and non-active recruiters concerning the demographic characteristics.

**Table 1:** Demographic characteristics of active and non-active recruiters

	**Total sample (*n*** **= 45)**	**Active (*n*** **= 12)**	**Non-active (*n*** **= 33)**	***t*-test**	***X***^**2**^	**df**	***p*-value**
Gender [% female (*n*)]	80.00 (36)	75.00 (9)	81.80 (27)		0.26	1	0.61
Age [M (*n*)]	41.60 (43)	43.50 (11)	40.90 (32)	0.85		23.6	0.41
Smoking [% smokers (*n*)]	4.70 (2)	0.00 (0)	6.30 (2)		1.44	3	0.70
Amount of hours every week [M (*n*)]	29.86 (44)	28.50 (12)	30.40 (32)	−0.95		42	0.35
Experience as HPP in years [M (*n*)]	3.42 (43)	3.64 (11)	3.34 (32)		1.05	3	0.79
Experience with smoke prevention in years [M (*n*)]	2.93 (43)	3.45 (11)	2.75 (32)		3.52	3	0.32
Experience within current MHPO in years [M (*n*)]	3.37 (43)	3.64 (11)	3.28 (32)		1.79	2	0.41
Total work experience in years [M (*n*)]	3.81 (43)	3.81 (11)	3.81 (32)		2.04	2	0.36
Familiar with Fun without Smokes [% yes (*n*)]	81.40 (35)	81.80 (9)	81.30 (26)		<0.01	1	0.97
Number of primary schools in region [M (*n*)]	269.95 (41)	260.28 (11)	273.53 (30)	−0.29		39	0.77

*n* = number of people.

M = mean.

### Differences between active and non-active recruiters

#### Psychological factors

Active recruiters had a significantly more positive *attitude* towards offering smoking prevention programmes to schools than non-active recruiters (*t*(41) = −2.35 (*p* = 0.02)). More specifically, active recruiters more strongly held the belief that recruiting schools for smoking prevention programmes is their task as HPP; they indicated being able to integrate the programme with their current methods, the ‘Fun without Smokes’ programme was not too complex to provide to schools in their region and they saw the advantages of the online and out-of-school design of the programme. *Social influences* were – overall – not significantly related to the decision to recruit primary schools actively. Yet, among active recruiters the manager and colleagues were significantly more supportive in the active recruitment than among non-active recruiters. Active recruiters had a higher score for *self-efficacy*, compared with non-active recruiters (*t*(33) = −4.87 (*p* < 0.01)). In depth analyses reveal, for example, that active recruiters were more confident in their ability to recruit and inform schools about the programme and they were able to make time to recruit primary schools for the ‘Fun without Smokes’ programme. Additionally, active recruiters were more willing to take *action* to inform and recruit primary schools for the ‘Fun without Smokes’ programme compared with non-active recruiters (*t*(38) = −5.33 (*p* < 0.01)). They planned, for instance, more often how to recruit schools actively, preserved more contacts of contacted schools and talked more with colleagues or superiors about the ‘Fun without Smokes’ programme, compared with non-active recruiters (Table 2).

**Table 2:** Means of and differences in psychological factors between active and non-active recruiters

**Variable (scale)**	**Total sample (*n*** **= 45)**	**Active (*n*** **= 12)**	**Non-active (*n*** **= 33)**	*p*-value*
***ATTITUDE *** **(agreement +2/-2)**	**0.70**	**0.92**	**0.60**	**0.02**
*I think….*				
…it is important to offer smoking prevention programmes to primary schools.	1.57	1.36	1.64	0.19
…it is a job of the MHPO to offer smoking prevention programmes to primary schools.	**1.02**	**1.45**	**0.88**	**0.04**
…it is my task to offer smoking prevention programmes to primary schools.	**0.41**	**1.27**	**0.12**	**0.01**
…that our smoking prevention programmes yield positive results.	0.44	0.27	0.50	0.33
…the programme ‘Fun without Smokes’ is an effective smoking prevention programme for primary school students.	0.49	0.27	0.56	0.14
…the programme ‘Fun without Smokes’ is an improvement compared with the current smoking prevention programme for primary school students.	0.60	0.82	0.52	0.28
…I can integrate the ‘Fun without Smokes’ programme within the current methods of the MHPO.	0.58	1.00	0.44	0.08
…it is a disadvantage it takes 1 hour to complete the ‘Fun without Smokes’ questionnaire^.	0.42	0.64	0.34	0.34
…the ‘Fun without Smokes’ programme is too complex to use in our region^.	**0.57**	**1.09**	**0.39**	**0.02**
…it is an advantage that ‘Fun without Smokes’ is an online and out-of-school programme.	0.67	1.00	0.56	0.12
*SOCIAL INFLUENCE* (agreement +2/-2)	<−0.01	0.18	−0.08	0.37
*Do you get support when you carry out a smoking prevention programme by…*				
…primary school teachers?	−0.37	−0.45	−0.33	0.80
…your manager?	−0.02	0.09	−0.07	0.74
…your colleagues?	0.24	0.27	0.23	0.94
…Trimbos Institute?	0.15	0.27	0.10	0.68
…Stivoro?	0.44	0.18	0.53	0.45
*Do you get help in the field of smoking prevention by…*				
…primary school teachers?	−0.80	−0.73	−0.83	0.77
…your manager?	−0.44	−0.36	−0.47	0.80
…your colleagues?	−0.12	0.09	−0.20	0.52
…Trimbos Institute?	<0.01	0.18	−0.07	0.56
…Stivoro?	0.10	0.27	0.03	0.58
*Do you think that…*				
…primary schools in your region will support the adoption of ‘Fun without Smokes’?	0.15	0.36	0.07	0.13
…your manager will support the adoption of ‘Fun without Smokes’?	**0.23**	**1.18**	**−0.14**	**<0.01**
…your colleagues will support the use of ‘Fun without Smokes’ within your region?	**0.35**	**1.00**	**0.10**	**<0.01**
***SELF-EFFICACY *** **(agreement +2/-2)**	**−0.11**	**0.62**	**−0.38**	**<0.01**
*Are you able to…*				
…offer smoking prevention programmes to primary schools in an active manner?	**0.17**	**0.91**	**−0.10**	**<0.01**
…execute smoking prevention programmes at primary schools?	0.05	0.55	−0.13	0.07
…convince primary schools of the importance of smoking prevention programmes?	0.26	0.27	0.26	0.96
…execute the programme ‘Fun without Smokes’?	**−0.15**	**0.64**	**−0.43**	**<0.01**
…offer ‘Fun without Smokes’ to primary schools in an active manner?	**−0.32**	**0.73**	**−0.70**	**<0.01**
…make your colleagues enthusiastic to use ‘Fun without Smokes’ within the current offers?	**0.29**	**0.82**	**0.10**	**0.01**
…inform primary schools about the goals and possibilities of ‘Fun without Smokes’?	**0.15**	**0.91**	**−0.13**	**0.01**
…make time to recruit primary schools for ‘Fun without Smokes’?	**−0.68**	**0.73**	**−1.20**	**<0.01**
…offer other smoking prevention programmes together with ‘Fun without Smokes’?	−0.56	−0.09	−0.75	0.15
…update the online database with contacted primary schools regularly?	**0.03**	**1.00**	**−0.34**	**<0.01**
…preserve contacts of contacted primary schools?	−0.18	0.36	−0.38	0.06
…recruit schools and classes for ‘Fun without Smokes’?	**−0.45**	**0.64**	**−0.86**	**<0.01**
***PREPERATORY PLAN *** **(agreement +2/-2)**	**0.01**	**1.19**	**−0.32**	**<0.01**
*I am planning to…*				
…visit the website of ‘Fun without Smokes’.	0.93	1.45	0.72	0.07
…talk about ‘Fun without Smokes’ with my manager.	**0.30**	**1.18**	**−0.03**	**<0.01**
…talk about ‘Fun without Smokes’ with my colleagues.	**0.73**	**1.36**	**0.48**	**0.01**
…search/ask for more information about ‘Fun without Smokes’.	0.30	0.64	0.17	0.24
…recruit primary school actively for ‘Fun without Smokes’.	**−0.30**	**1.64**	**−1.03**	**<0.01**
…preserve the contacts of the contacted primary schools.	**−0.40**	**0.82**	**−0.86**	**<0.01**
…update the database of ‘Fun without Smokes’ regularly.	**−0.33**	**1.18**	**−0.90**	**<0.01**
…ask colleagues for assistance to recruit primary schools for ‘Fun without Smokes’.	**−0.45**	**1.27**	**−1.10**	**<0.01**

Significant *p*-values are marked bold.

*p-values are for t-tests comparing active and non-active recruiters.

^variable recoded (−2/+2).

#### Organisational factors

Table 3 depicts that no significant differences between active and non-active recruiters were found as regards the *decision-making style* within the MHPOs. Active recruiters indicated having more *decision power* and more *time for smoking prevention* programmes compared with non-active recruiters, but these effects were not significant.

**Table 3:** Means of and differences in organisational factors between active and non-active recruiters

**Variable**	**Total sample (*n*** **= 45)**	**Active (*n*** **= 12)**	**Non-active (*n*** **= 33)**	*p*-value*
*DECISION-MAKING* (agreement +2/-2)				
Centrality	1.26	1.50	1.17	0.12
Formalisation	0.21	<0.01	0.30	0.32
Information	1.29	1.17	1.30	0.39
Confrontation	−0.20	−0.08	−0.24	0.59
*TIME FOR SMOKING PREVENTION* (agreement +2/-2)				
Do you have enough time to offer smoking prevention programmes to primary schools?	−0.15	0.27	−0.31	0.11
*DECISION POWER* (agreement 1 – 4)				
How big was your share in the decision whether or not to take part in the active recruitment of primary schools with the smoking prevention programme ‘Fun without Smokes’?	2.88	3.09	2.80	0.48

*p-values are for t-tests comparing active and non-active recruiters.

### Multivariate analyses

In order to assess the strength of the relation between psychological and organisational factors, on the one hand, and the decision to recruit primary schools, on the other, multivariate analysis of variance was performed. These analyses reveal that psychological factors are more strongly related to the participation of MHPOs in the active recruitment of primary schools (η^2^ = 0.44 (*p* < 0.01)) than organisational factors (η^2^ = 0.21). The difference between these two effect sizes appeared to be significant (95% BCa-CI: 0.05 – 0.54). Attitude (η^2^ = 0.13 (*p* = 0.02)), self-efficacy (η^2^ = 0.26 (*p* < 0.01)) and preparatory plans (η^2^ = 0.43 (*p* < 0.01)) were the psychological factors that differed most between active and non-active recruiters.

## Discussion

### Main results

The aim of the present study was to assess psychological and organisational factors associated with the willingness of Dutch MHPO employees to participate in the active recruitment of primary schools to use a smoking prevention programme. The results showed that psychological factors were more strongly related to active recruitment behaviour than organisational factors. Compared with non-active recruiters, active recruiters had a more positive attitude and higher self-efficacy expectations regarding the active recruitment of primary schools. Furthermore, active recruiters formulated significantly more preparatory plans than non-active recruiters.

Even though studies on factors that may influence active recruitment or dissemination behaviour of intermediary agents or organisations are rare, the results of this study are in line with those of other studies that have investigated dissemination and implementation of evidence-based practices in intermediary organisations and also found a positive attitude to be important in the adoption of these interventions [28,29]. Similar to our study, Segaar and colleagues [20] reported that cardiac nurses who adopted a Minimal Intervention Strategy for smoking cessation in cardiac inpatients (adopters) had a more positive attitude and higher self-efficacy expectations compared with non-adopters. The results of the present study showed that attitudes play an important role in the decision whether or not to participate in the active recruitment of primary schools and active recruiters had more positive beliefs than non-active recruiters. However, not every HPP has a positive attitude towards the promotion and diffusion of a smoking prevention programme. This is quite concerning for an organisation that is legally responsible for the dissemination of smoking prevention programmes to schools and may be reason for further investigation. Besides HPPs’ beliefs, it was also a striking finding that only seven out of 31 Dutch MHPOs were prepared to participate in the active recruitment procedure, which stress the importance of studying this topic. Furthermore, the finding that preparatory plans are important in a dissemination process is also in line with that in previous studies [10,30,31], which revealed a positive influence of planning on the adoption and implementation of health promotion programmes or activities. Our results showed that active recruiters formulated more plans to talk with colleagues and their superior about the dissemination of a smoking prevention programme. HPPs also need the support of these people to recruit primary schools actively. In the analyses, social influences were not related to the decision to recruit primary schools. However, specifically, the social support expected from colleagues and the superior were significantly related to the dissemination of the ‘Fun without Smokes’ programme among the active recruiters. In previous studies it has been reported that a social system (colleagues, superiors or opinion leaders) can both have a positive or a negative influence on decisions (concerning adoption or innovation) within an organisation [17,32]. The results in the present study suggest that consultation with colleagues or superiors has a positive influence concerning the participation in a recruitment procedure. However, to investigate accurately this consultation process measures other than those assessed in this study might be needed.

No association was found between organisational factors and active recruitment. Active recruiters reported that they had more decision power than non-active recruiters, but this relation was not significant. A possible explanation that no significant association was found between active and non-active recruiters might be that other organisational factors than those investigated in the present study, such as policy regarding smoking prevention, innovativeness or financial resources, play a significant role. Rogers [17] also stated the importance of organisational factors in an innovation decision-making process. However, in most cases, an individual cannot adopt a new idea or innovation until an organisation has previously adopted it. The majority of the decisions among active recruiters in the present study were made by health promotion officers who have to execute the active recruitment of primary schools. For that reason, it seems to be more efficient to contact the subordinates (such as a health promotion officer) and not the higher managers, since they have the power to make decisions whether or not to participate in recruitment procedures.

### Strengths and limitations

Measuring both the psychological and organisational factors is a strength of the present study. This is also supported by other studies, since both factors can affect the degree to which an innovation is disseminated [20,33]. An additional positive remark is that all Dutch MHPOs, including all HPPs who focus on smoking prevention in Dutch schools, were contacted and that most of the MHPOs participated in the study, which improves the generalisability of findings. The aim of the present study was to identify factors associated with the dissemination of a smoking prevention programme to schools. However, this study was also concerned with the recruitment of primary schools to participate in an evaluation study. Strictly speaking, this is not an implementation study, nevertheless, the findings may also relate to the dissemination of an intervention programme only. A difference might be that in the present study the effectiveness of the intervention is not yet demonstrated. A second limitation might be that due to the fact that the questionnaire was based on self-report, it might be plausible that socially desirable answers were given, since the answers in the questionnaire will be a representation of the MHPO where the HPP is employed. Finally, characteristics of the organisational context in MHPOs were not measured in detail in the present study; this makes it difficult to make statements about the innovativeness, availability of resources (e.g. finances) or the policy concerning the dissemination of smoking prevention programmes of the individual MHPOs. Other studies support the inclusion of extended concepts to measure organisational characteristics [32,34,35]. Though the organisational factors used in this study give an overall view of the factors related to the decision-making process of the MHPOs, it is advisable to investigate these organisational characteristics in more depth in future research.

## Conclusions

Although this study was subject to several limitations, it can be concluded that primarily psychological factors seem to have an impact on the decision of MHPOs to participate in active recruitment of primary schools to use a web-based smoking prevention programme. A positive attitude and self-efficacy expectations and creation of specific plans for how and when to perform recruitment activities were associated with active recruitment of schools. This indicates that creating a more positive attitude, self-efficacy beliefs and formation of plans may help in getting more MHPOs and HPPs involved in active recruitment. In future research, it may be important to investigate whether more specific organisational factors such as policy regarding dissemination of smoking prevention programmes to schools or norms and culture of MHPOs have an influence on the decision whether or not to participate in the active recruitment of schools. Furthermore, it is recommended to encourage decision makers to form plans to take action or provide them with specific plans to be more involved in the recruitment procedure. To get MHPOs prepared to participate in the dissemination of prevention programmes, it seems efficient to approach HPPs who are most involved in executing the recruitment, since they have most decision power whether or not to participate.

## Competing interests

HdV is scientific director of Vision2Health, a company that licenses evidence-based innovative computer-tailored health communication tools. The other authors declare that they have no competing interests.

## Authors’ contributions

HdV designed and wrote the original proposal. HPC, AO, LM and HdV designed and executed the present study. MC advised on the execution of the statistical analyses. HPC significantly contributed to writing the present article, while AO, LM, MC and HdV were involved in revising the manuscript critically. All authors read and approved the final manuscript.
